# C10Pred: A First Machine Learning Based Tool to Predict C10 Family Cysteine Peptidases Using Sequence-Derived Features

**DOI:** 10.3390/ijms23179518

**Published:** 2022-08-23

**Authors:** Adeel Malik, Nitin Mahajan, Tanveer Ali Dar, Chang-Bae Kim

**Affiliations:** 1 Institute of Intelligence Informatics Technology, Sangmyung University, Seoul 03016, Korea; 2 Department of Pediatrics, Washington University in St. Louis, St. Louis, MO 63110, USA; 3 Department of Clinical Biochemistry, University of Kashmir, Srinagar 190006, India; 4 Department of Biotechnology, Sangmyung University, Seoul 03016, Korea

**Keywords:** C10 family, cysteine peptidase, streptopain, machine learning, support vector machine, feature selection, Boruta

## Abstract

*Streptococcus pyogenes*, or group A *Streptococcus* (GAS), a gram-positive bacterium, is implicated in a wide range of clinical manifestations and life-threatening diseases. One of the key virulence factors of GAS is streptopain, a C10 family cysteine peptidase. Since its discovery, various homologs of streptopain have been reported from other bacterial species. With the increased affordability of sequencing, a significant increase in the number of potential C10 family-like sequences in the public databases is anticipated, posing a challenge in classifying such sequences. Sequence-similarity-based tools are the methods of choice to identify such streptopain-like sequences. However, these methods depend on some level of sequence similarity between the existing C10 family and the target sequences. Therefore, in this work, we propose a novel predictor, C10Pred, for the prediction of C10 peptidases using sequence-derived optimal features. C10Pred is a support vector machine (SVM) based model which is efficient in predicting C10 enzymes with an overall accuracy of 92.7% and Matthews’ correlation coefficient (MCC) value of 0.855 when tested on an independent dataset. We anticipate that C10Pred will serve as a handy tool to classify novel streptopain-like proteins belonging to the C10 family and offer essential information.

## 1. Introduction

*Streptococcus pyogenes*, or group A *Streptococcus* (GAS), a gram-positive bacterium, is a reason for causing a wide range of clinical manifestations, from superficial infections to severe, life-threatening diseases [1,2]. Globally, GAS pharyngitis accounts for more than 600 million cases annually [3]. Another superficial infection frequently caused by GAS is impetigo, commonly found in tropical, resource-poor settings [4]. It has been estimated that more than 162 million children globally suffer from impetigo at any time [5]. On the other hand, GAS causes severe and life-threatening infections, such as bacteremia, meningitis, necrotizing fasciitis (NF), sepsis, and streptococcal toxic shock syndrome (STSS) [6,7]. Furthermore, GAS infection can also develop immune-mediated sequelae like acute rheumatic fever (ARF) and acute post-streptococcal glomerulonephritis (APSGN) [8]. ARF can lead to rheumatic heart disease (RHD), a significant cause of mortality and morbidity resulting from the severe condition associated with cardiac failure, stroke, and early death [8]. APSGN can contribute to chronic renal failure [9,10]. Although now rare in developed world countries, ARF and APSGN still maintain a significant presence in economically disadvantaged populations accounted for by poor hygiene and limited resources [11,12]. However, relatively rare invasive infections are often associated with high mortality and morbidity [3]. The global incidence of invasive GAS diseases is reportedly around 6 cases per 100,000 people per year [13]. The highest incidence of invasive GAS diseases is seen in the elderly, followed by young children, particularly those under one year of age [14]. GAS infections are significantly associated with high morbidity and mortality rates worldwide. GAS diseases were estimated to cause more than 500,000 deaths in 2005 [3], and RHD alone was estimated to account for 320,000 deaths in 2015 [15].

Streptopain, also known as streptococcal pyrogenic exotoxin B (SpeB), streptococcus peptidase A (SPP), and streptococcal cysteine protease (SCP), is one of the major virulence factors for GAS [16,17,18]. According to MEROPS [19], an online peptidase database, streptopain is classified as a C10 family (also known as the streptopain family) of cysteine peptidases. There are 16 clans of cysteine peptidases, including some unclassified, of which four comprises proteases with diverse catalytic types [19]. Each of these clans exhibits a distinct fold and is further divided into separate families [20]. Streptopain belongs to Clan CA of cysteine proteases and shares the clan with the first-ever discovered cysteine protease, i.e., papain (isolated from *Carica papaya*). Cysteine proteases use the reactive site cysteine as the catalytic nucleophile and the histidine to perform peptide bond hydrolysis. Streptopain shares limited similarity with papain, and the catalytic cysteine and histidine residues in streptopain (C47and H195) have similar order to papain (C25 and H159), including some identical neighboring residues [19,20]. Sequence analysis of all intermediates and final product demonstrates that streptopain, similar to papain, prefers substrates with hydrophobic residues [21]. However, streptopain lacks the presence of Asn or Asp residue equivalent to the Asn in the papain family, which forms the catalytic triad in cysteine peptidases. Also, in contrast to other papain family members, streptopain has significant insertions and deletions outside its conserved core. The pro-domain of streptopain has a fold that is unique among the other proteases [22].

Streptopain is present in all the isolates of *S. pyogenes*, and therefore is a predominant extracellular protein that accounts for approximately 95% of total secreted proteins [23]. Streptopain is extracellularly released from GAS to the culture medium in a zymogen form, i.e., proSPEB [24,25]. Zymogen (proSPEB) is converted to the mature mSPEB either by autoproteolysis or exogenous proteases. Structurally, the conformation of the C-terminal loop and the orientation of the catalytic H195 residue plays an important role in activating proSPEB to mSPEB [21]. NMR analysis demonstrates that the C-terminal loop of streptopain is flexible, controls the substrate binding, and therefore has diverse substrate specificity [22,26]. Streptopain has diverse substrate specificity in processing streptococcal proteins and host proteins. This diverse substrate specificity leads to its different biological effects [27]. For example, streptopain degrades extracellular matrix (ECM) proteins fibronectin and vitronectin, which help in bacterial attachment to host cells [28]. It also cleaves and activates matrix metalloproteases (MMPs) and therefore helps in extracellular matrix degradation, which eventually leads to increased bacterial dissemination [29,30]. In addition to host proteins, streptopain releases streptococcal surface proteins like M-protein, protein F1, protein H, Sda1, Fba, and superantigen1 [27]. Besides protease activity, streptopain also exhibits transferase and esterase activities. A variant of streptopain with an Arg-Gly-Asp motif that binds integrins αvβ3 and αIIbβ3 has been reported in the M1 serotype isolates [31].

Although much of the work on this C10 cysteine peptidase has been reported from Streptococcal strains, studies have also identified SpeB homologs from other bacterial species. Among them is interpain A (InpA), which was identified from an oral anaerobe *Prevotella intermedia*. InpA plays an essential role in the oxidation and breakdown of hemoglobin and the subsequent release of haem [32]. Similarly, two genes encoding periodontain (PdnA) and thiol protease/hemagglutinin (PrtT) from *Porphyromonas gingivalis* share significant homology to SpeB [33]. Streptopain homologs have also been discovered from bacterial species that inhabit organisms other than humans. This includes bacteria that are pathogenic to marine aquaculture. For example, the *FcpB* gene in *Flavobacterium psychrophilum*, a Gram-negative fish pathogen, encodes a 394 amino-acid protein fcpB [34]. This protein shares significant homology with cysteine peptidases such as streptopains and other C10 family members from different bacterial species, including *Flavobacterium branchiophilum*, *Dyadobacter fermentans*, *Bacteroides intestinalis,* and *Spirosoma linguale* [34]. Similarly, a gene cluster *MARIT_2328* in *Tenacibaculum maritimum* encodes a multi-domain protein from the C10 family peptidase, which is significantly similar to SpeB, and likely plays a role in colonization and invasion [35]. The genomic overview of the peptidases of anaerobic Gram-negative bacteria *Prevotella* and *Paraprevotella* species which inhabit the oral cavity, GI tract, and urinary tract of animals and humans, provided a comprehensive analysis of various peptidases [36,37]. Genomic sequencing of *Prevotella* and *Paraprevotella* species demonstrated the presence of a total of 78 distinct peptidase families. This analysis shows that C10 family peptidases were among the most abundant [38].

Since its discovery, several streptopain homologs have been identified, many of which remain uncharacterized. Additionally, the large-scale bacterial genome sequencing projects may have led to a rapid increase in the number of potential C10 family-like sequences in the public databases, posing a challenge to annotating such sequences. Moreover, experimental identification and characterization of streptopain and its homologs is costly and time-consuming. Therefore, novel computational methods are required that provide robust techniques for correctly identifying C10 family cysteine peptidases from their primary amino acid sequences. At present, methods based on sequence-similarity such as BLAST [39] and HMMER [40] are the only approaches that are available to identify streptopain-like sequences. However, one of the main drawbacks of such techniques is that they are meaningful only if there exists some level of sequence similarity between the existing C10 family and the target sequences. Consequently, these methods fail to discover novel sequences comprising streptopain-like domains. Hence, machine learning (ML)-based approaches offer encouraging alternatives to develop novel predictors for such classification problems.

With this background, we propose the first ML-based tool, C10Pred, which can predict C10 family proteins from their primary sequences. The predictor incorporates optimal features from different encodings (hybrid features) for better performance. We expect that C10Pred will be a competent tool for identifying the C10 family or streptopain-like sequences, which will help investigate their functional roles in many bacterial diseases. The C10Pred web server is freely available at https://procarb.org/c10pred/ (accessed on 16 August 2022).

## 2. Results

### 2.1. Overview of the Dataset

**Figure 1:**
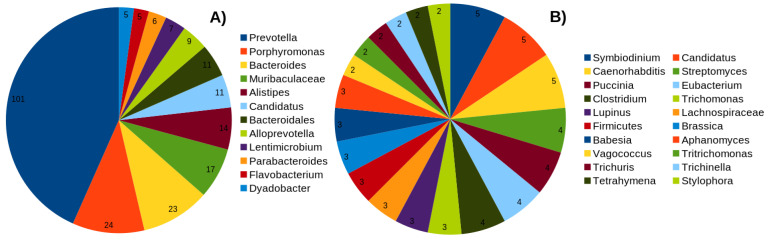
Number of most abundant taxonomic groups in the (**A**) positive, and (**B**) negative datasets. Complete list is provided in Table S1.

The positive dataset comprises a non-redundant set of 336 C10 family peptidases belonging to the PFAM family “Peptidase_C10”, whereas the negative data consists of 350 sequences from other cysteine peptidase families within the MEROPS [19] database (Table 1). This dataset represents sequences from a wide range of taxonomic groups and corresponds to 82 and 283 unique bacterial taxonomic groups in the positive and negative datasets, respectively (Figure 1 and Table S1). The functional annotation of the dataset was carried out with an eggNOG mapper [41] and suggested that more than 80% of sequences in the positive dataset belong to an unknown functional category (S = 43.54%) or did not have any hits in the eggNOG database. In contrast, the annotation was much better for the negative dataset. Some of the well-annotated COG categories include posttranslational modification, protein turnover, chaperones (O = 18.85%), cell wall/membrane/envelope biogenesis (M = 12%), and amino acid transport and metabolism (E = 8.57%) (Figure S1). These data suggest that only limited information is available on the functional roles of C10 family peptidases and their homologs.

### 2.2. The Overall Framework of the Proposed Predictor

**Figure 2:**
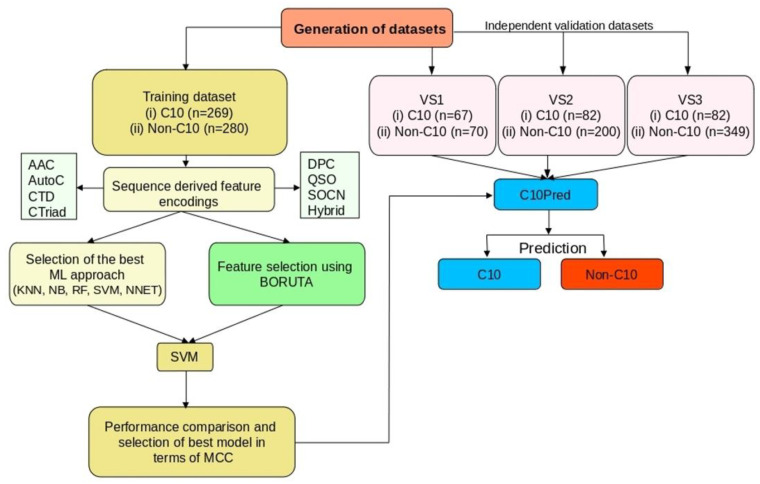
Schematic overview of the C10Pred tool demonstrating the four main stages of the predictor development. The first stage comprises the generation of the datasets, and the second stage consists of feature extraction from the primary amino acid sequences. In the third stage, we constructed five ML-based classifiers, namely, KNN, NB, RF, SVM, and NNET, using different feature sets and selected the best classifier. In parallel, we also performed feature selection using the Boruta algorithm. Finally, SVM was selected as the best ML classifier, and the performance of various optimal feature encodings was evaluated. KNN: K-nearest neighbors; NB: Naive Bayes; RF: random forest; SVM: support vector machines; NNET: neural network.

Figure 2 summarizes the overall framework for the C10Pred, which essentially consists of four main steps: (i) construction of training and independent datasets; (ii) encoding of various sequence-derived features (e.g., AAC, AutoC, CTD, CTriad, DPC, QSO, SOCN, and Hybrid); (iii) feature selection using Boruta algorithm, and (iv) selection of the final model exhibiting best performance in terms of MCC. Accordingly, the corresponding feature set with the best performance was considered to be the optimal set.

### 2.3. Amino Acid Composition in C10 and Non-C10 Sequences

**Figure 3:**
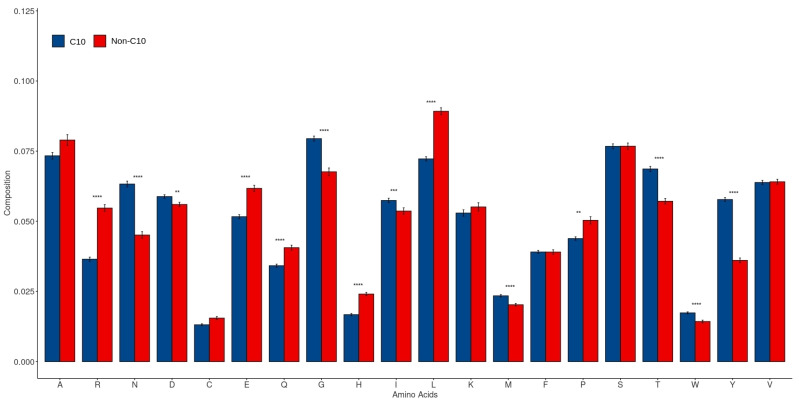
Average amino acid composition (AAC) difference between C10 and non-C10 family sequences. Asterisks above the bars indicate the *p*-value (** = *p* < 0.01; *** = *p* < 0.001; and **** = *p* < 0.0001).

To determine the presence of any compositional differences between C10 and non-C10 peptidases, we compared the AAC of the positive and negative datasets. The AAC of both these datasets is shown in Figure 3, which shows the higher frequency of hydrophobic amino acids like tryptophan (W), methionine (M), glycine (G), Isoleucine (I), and uncharged polar amino acids, including asparagine (N), threonine (T), and tyrosine (Y) in the C10 protein sequences (Wilcox test; *p* < 0.05) as compared to non-C10 peptidases. Interestingly, as compared to C10 peptidases with only aspartic acid as the dominant polar residue, the non-C10 were dominant in most of the charged polar residues, including arginine (R), glutamate (E), and histidine (H) residues. The most important characteristic feature for identification purposes could be the lesser frequency of charged polar residues and dominance of hydrophobic amino acids, particularly tryptophan, the rarest of the amino acids. The dominance of hydrophobic amino acids with a lesser frequency of charged residues in turn signifies the increased stability of C10 peptidases compared to non-C10 peptidases. The peculiar compositional differences between the peptidases in turn infer that our model could use the presence of specific amino acids as a suitable strategy to categorize C10 peptidases from non-C10 peptidases.

### 2.4. Comparison of Various Machine Learning Classifiers

**Figure 4:**
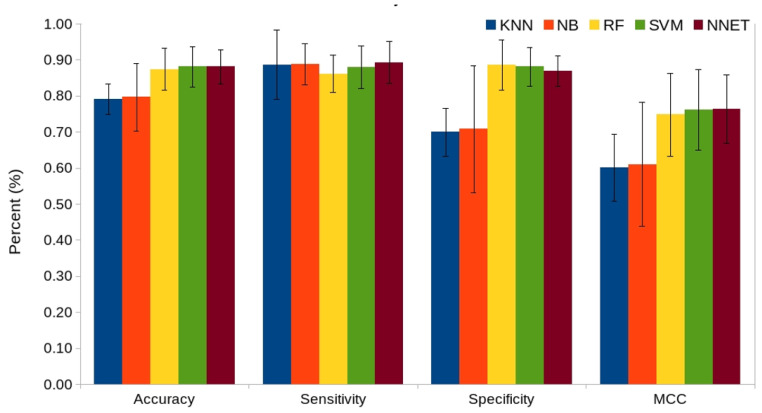
Average performance comparison of five ML-based classifiers (KNN, NB, RF, SVM, and NNET) using eight different feature encodings. The performance of each individual feature encoding for all classifiers is shown in Figure S2.

To assess the performance of various ML classifiers, we exploited five commonly used ML approaches (KNN, NB, RF, SVM, and NNET) on seven independent feature encodings and their hybrid. The performance of all these classifiers was assessed by using 10-fold cross-validation. Our comparative analysis suggests that the average performance of SVM was consistently better than four other classifiers in terms of accuracy and MCC on multiple feature encodings (Figure 4 and Figure S2). Although the average performance of NNET was equally better, SVM demonstrated a slight edge by performing better on 4/8 descriptors (e.g., AAC, AutoC, CTD, and CTriad). In contrast, DPC, SOCN, and hybrid feature encodings performed better when NNET was used to train the model. Similar performance was observed for both these models when QSO was used as an input feature. These data indicated that SVM was the best performing classifier, and thus it was selected for further analysis.

### 2.5. Performance Evaluation of Various Feature Encodings

We used SVM to probe the potential of each feature encoding in correctly differentiating C10 and non-C10 peptidases using 10-fold cross-validation. The performance achieved by each descriptor is shown in Table 2. Our data on the 10-fold cross-validation test shows that DPC followed by hybrid features achieved the best performance with accuracy scores of 93.4% and 92.9%, respectively. In addition to the high accuracy, these two features also exhibited encouraging MCC values that ranged between 0.85–0.86. In contrast, the accuracy scores for other descriptors (AAC, AutoC, CTD, CTriad, and QSO) were reasonable (84–90%), although with limited MCC scores. Models based on SOCN were the worst performing, with an accuracy of 75% and an MCC of 0.508.

### 2.6. Optimal Feature Selection for Each Encoding

Recognizing that almost all the features except AAC and SOCN have large dimension sizes (≥100), some of the encodings might be superfluous or may not be equally significant. Therefore, this necessitates the application of feature selection protocol to eliminate redundant and insignificant encodings. We applied the Boruta algorithm to explore if it was able to slash the feature dimensions and affect the overall performance. Table 3 compares the performance achieved by various feature encodings using optimal features when classifying C10 and non-C10 peptidases. From this table, we also observed that when predicting C10 and non-C10 peptidases, the number of features was significantly reduced for hybrid features (92.23%), AutoC (85.83%), CTriad and DPC (~80%), QSO (55%), and CTD (39.45%). A limited number of features (3.33%) were removed for SOCN, while no dimension reduction was observed for AAC.

**Figure 5:**
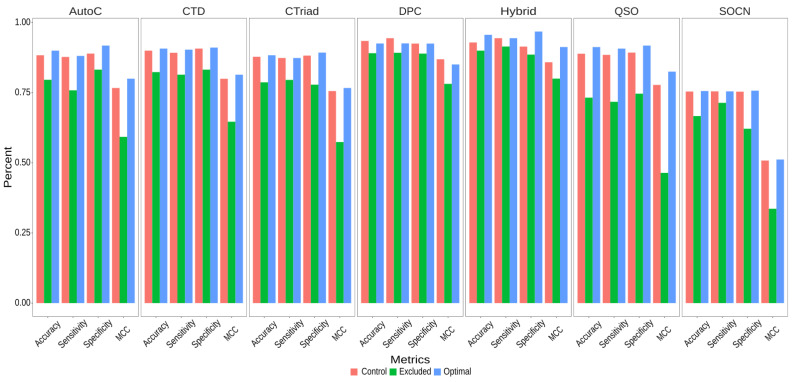
Performance comparison of SVM-based models using all features (control), excluded features and optimal features.

Following the reduction of feature dimensions, we explored the performance of each feature encoding using the optimal features and compared it with the respective controls (all features). Figure 5 shows a marginal improvement in the performance of most feature encodings, especially in the AutoC, QSO, and the hybrid, by 1.64%, 2.37%, and 2.73%, respectively, as compared to their controls. The improvement shown by CTD, CTriad, and SOCN is only marginal (<1%). Interestingly, there was a slight decrease of 0.01% in the performance of DPC when optimal feature sets were used.

Next, to examine whether the optimal features are any better than the features excluded for each feature encodings, we developed prediction models based on excluded features and compared their performance with the control (using all features) and the optimal features. We observe that the models based on optimal features performed consistently better than those based on the excluded features (Figure 5). Notably, the average accuracy achieved by the optimal feature-based models is about 10% higher than the models based on excluded features and 1% higher compared to the controls when predicting C10 peptidases. Similarly, models based on optimal features exhibit better MCC scores than the control and excluded features. For example, using control feature encodings for AutoC, QSO, and hybrid, the classifier exhibited the MCC scores of 0.767, 0.778, and 0.858, respectively. However, using optimal features for these encodings, a significant increase in their MCC scores was observed (e.g., 0.80, 0.825, and 0.913). In contrast, classifiers based on excluded features performed worst, and the MCC scores for these three encodings are 0.593, 0.464, and 0.80, respectively. These data suggest that the Boruta algorithm identified important features contributing to improved performance and overall dimension reduction.

### 2.7. Performance Comparison on Independent Datasets

**Figure 6:**
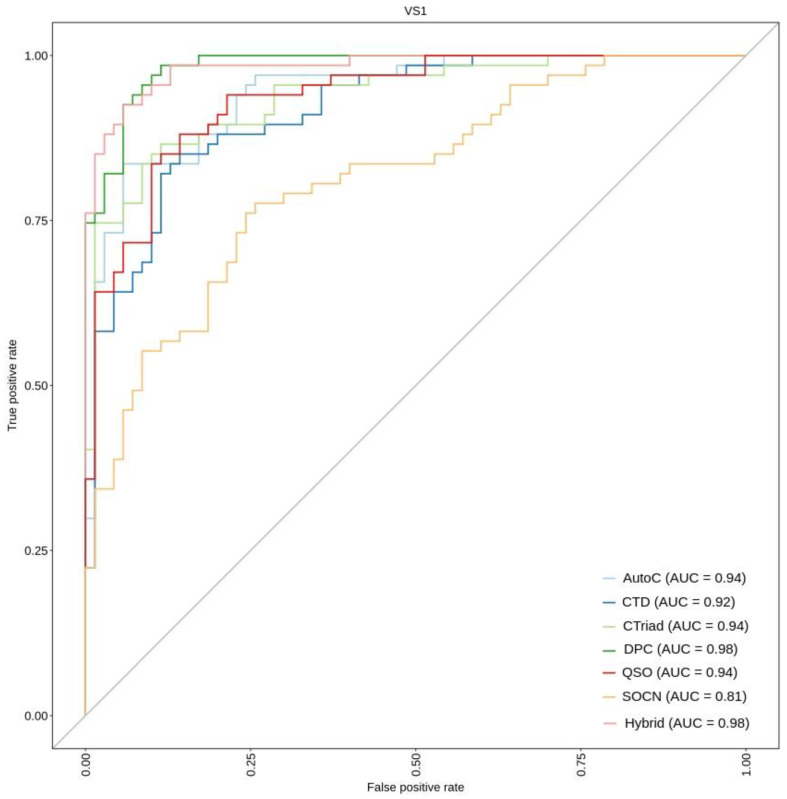
Comparison of binormal receiver operating characteristics (ROC) curves for various prediction models on the independent dataset VS1. Higher scores indicate better performance of that specific model.

It is well known that the testing of an ML algorithm on the training data does not provide the best clue regarding its performance on the unseen data because of the deceivingly overall high accuracies [42]. Therefore, to verify whether the consistence performance is shown by various feature encodings, we assessed each of these optimal feature-based encodings on the independent validation set VS1. The results on VS1 indicate that hybrid features show the best performance, which is similar and consistent with the performance obtained in the 10-fold cross-validation test (Table 3 and Table 4). This hybrid model using optimal features achieves an accuracy, sensitivity, specificity, and MCC of 0.927, 0.896, 0.957, and 0.855, respectively. Specifically, the accuracy and MCC achieved using hybrid encodings are approximately 3–19% and 7–38% higher, respectively, than the other feature encodings. Furthermore, to graphically visualize the performance of various encodings, an ROC curve was generated by computing and plotting the true positive rate (TPR) versus the false positive rate (FPR) (Figure 6). In such plots, a higher AUC score indicates a better classifier performance. From this figure, we again observe that the hybrid classifier using optimal features showed the best AUC of 0.98. These data demonstrate that the hybrid model using optimal features has the potential to accomplish promising performance. Therefore, this classifier was selected as a final model. Although DPC based classifier also exhibited a similar AUC value (Figure 6), it showed poor performance when other evaluation metrics such as accuracy and MCC were considered.

**Figure 7:**
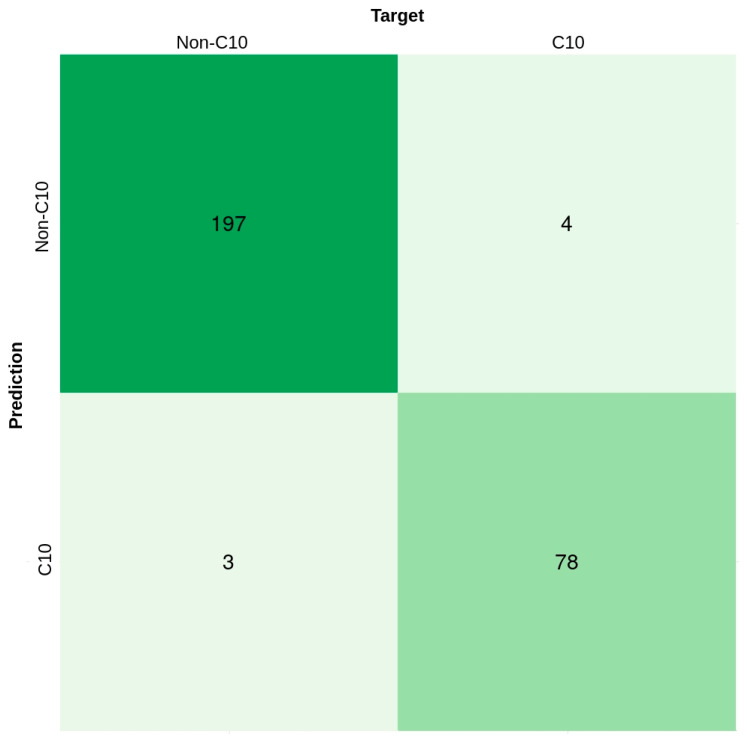
Confusion matrix of predicted results on additional independent dataset VS2. The matrix represents the output distribution for each of the two classes (C10 or non-C10).

To further assess the performance of C10Pred we used an additional validation set, VS2. Figure 7 shows the confusion matrix for predicting the binary classification of C10 peptidases. Specifically, the figure shows that 4/82 positive sequences were incorrectly predicted, whereas only 3/200 negative sequences were classified as positive sequences. The three negative sequences, incorrectly predicted as positive sequences, include papain domain containing C1 family cysteine protease, caspase P20 domain-containing protein, and a C25 family cysteine protease, respectively. Interestingly, similar to the amino acid composition of the positive dataset (Figure 3), all these three sequences also exhibited a higher percentage of TYR residue than the average value for the negative sequences. Similarly, the ¾ positive sequences, wrongly identified as negative sequences, exhibited a TYR profile similar to the negative sequences. The amino acid composition of some other residues (e.g., TRP, LEU, and GLY) in these four positive sequences also deviated and was analogous to their counterparts in the negative dataset. The accuracy, MCC, and AUC achieved by our proposed method on the VS2 dataset are 0.975, 0.94, and 0.968, respectively. As mentioned in the methods section, the negative dataset comprises sequences representing all other cysteine peptidases except C10 family proteases. Therefore, to assess how the model behaves if a more diverse set of sequences is used as a negative dataset rather than just other families of cysteine proteases, we compiled another non-redundant dataset of 349 negative sequences from the UNIPROT [43] database. This new set of negative sequences was merged with the positive sequences of the VS2 dataset to form additional validation set VS3 (Table 1). Compared to VS1 and VS2, VS3 consists of all bacterial sequences except the C10 family or streptopain proteins. It should be noted that both VS2 and VS3 validation sets comprise sequences that show <50% sequence similarity with the positive data. On assessing the performance of C10Pred using the VS3 dataset, a slight increase in accuracy (0.979%), with a small decrease in MCC (0.933), was observed (Figure S3). Altogether, the results demonstrate the remarkable performance achieved by C10Pred, which could be further enhanced by exploiting large-scale training data when it becomes available in the future.

### 2.8. Software Availability

To make our method publicly available so that potential users may benefit from it, both the standalone as well as the webserver version for C10Pred are freely accessible at the following link: https://procarb.org/c10pred/ (accessed 16 August 2022). The input to both the versions is the fasta formatted sequences, and the prediction results are available as a downloadable comma-separated file (CSV). All instructions and datasets used in this work are available on the C10Pred homepage. 

## 3. Discussion

*S. pyogenes* expresses a highly conserved virulence factor streptopain (a C10 family cysteine peptidase) known to degrade an array of GAS and host proteins [44]. Although much of the work on this C10 cysteine protease has been reported from *Streptococcal* strains, many recent studies have identified this protease or its homologs in other bacterial species [34,35,45,46]. The biological activities and molecular functions of proteins can be predicted from their amino acid sequences [47]. Therefore, in the present study, we exploited the available C10 family sequences to develop the ML-based predictor, C10Pred, to classify the C10 family proteases.

We utilized seven feature encodings (AAC, AutoC, CTD, CTriad, DPC, QSO, and SOCN) and also combined all these features (hybrid) to predict C10 proteases using SVM. These features have been used extensively in various sequence-based protein classification problems [48,49,50,51,52]. The performance results from each encoding performed well, especially with DPC and CTD-based classifiers. However, the predictor’s performance using SOCN descriptors was moderate. Interestingly, when we applied the feature selection protocol, we observed that optimal hybrid encodings outperformed the other features. Therefore, we considered it to be the most efficient feature for the prediction of C10 enzymes. This dimension reduced the optimal hybrid feature set of 139 descriptors showing a >2% increase in the overall accuracy of the classifier and about a 5% increase in its MCC score. The corresponding sensitivity and specificity values for these optimal 139 features are 0.944 and 0.968, respectively. Selection of optimal features is one of the essential steps in developing ML-based models because the original set may contain redundant and non-informative features [49]. These copious non-informative and redundant descriptors, especially in the case of high dimensional features, affect the prediction accuracy. Therefore, selecting optimal features is regarded as one of the most influential steps in ML-based prediction [53,54,55,56]. Recognizing the potential of feature selection, we applied the Boruta feature selection method, which has been widely applied effectively in several biological applications [57,58,59], and consequently identified optimal features. Among these, the major contribution was from AutoC (~27%), followed by CTD, DPC, QSO, CTriad, AAC, and SOCN. Although AutoC descriptors were the major contributors, the top 10 most important features were dominated by DPC (5/10), QSO (4/10), and a single Y residue of AAC. These top-scoring dipeptides included GW, GC, WG, GY, and YN. It should be noted that these dipeptides comprise residues that are more abundant in C10 peptidases than non-C10 peptidases (Figure 3).

Based on the performance obtained on the hybrid model using an optimal feature set, the SVM-based predictor C10Pred was constructed. Moreover, the dataset generated in this work has a stringent sequence identity of ≤40%, which is essential to avoid overestimating the predictive performance of a predictor [49]. Importantly, this is the first ML-based method for predicting C10 family peptidases using sequence-derived information, and is freely available as a web server. Since there is no other method available for the prediction of C10 peptidases or their homologs, a direct comparison is impossible. Although C10Pred exhibited acceptable predictive performance, there is scope for further improvements. For example, constructing a model on a larger dataset when it becomes available, testing other feature encodings, and exploiting different ML algorithms such as stochastic gradient boosting [60].

## 4. Materials and Methods

### 4.1. Data Acquisition and Data Organization

All protein sequences representing “the Peptidase_C10” family (PFAM ID: PF01640) within the PFAM [61] database were retrieved. All non-standard amino acids containing sequences were removed, and sequences shorter than 100 amino acids were also excluded. The remaining sequences were subjected to a redundancy removal by applying CD-HIT v4.8.1 [62] with the 40% sequence identity cut-off.

The negative dataset was generated as follows: (i) retrieved all PFAM sequences belonging to various cysteine peptidase clans/families except the family C10 which was used as a positive dataset. (ii) Non-standard amino acids containing sequences were eliminated. (iii) Sequences having a length between 100 and 2300 amino acids were retained only, and (iv) We further filtered the negative dataset at 40% sequence identity cut-off using CD-HIT. These steps generated a large number of over 47,000 sequences in the negative dataset. To generate a balanced dataset, we randomly selected negative samples that were similar in number to the positive dataset. To ensure a limited similarity between the positive and negative datasets, we removed all the negative samples that showed ≥25% sequence identity with the positive dataset.

Both these datasets mentioned above were combined and divided into a training and an independent validation set (VS1) by using the createDataPartition function of the CARET (short for Classification And REgression Training) package [63] available in R (https://www.r-project.org/: accessed 16 August 2022).

Furthermore, to assess the robustness of our method, we constructed an additional independent validation set (VS2) by retrieving all the streptopain sequences available in the NCBI protein database. After filtering non-standard amino acid-containing sequences, the sequences were further processed for redundancy removal at a 50% sequence identity cut-off. Subsequently, we removed all the sequences that shared ≥50% sequence similarity with the positive dataset. Again, a negative dataset of 200 sequences was randomly constructed and combined with these sequences. The overall summary of the datasets is provided in Table 1.

### 4.2. Feature Encoding

To develop an ML model, sequences with varying lengths were converted to fixed-length feature vectors using feature encoding algorithms. In this work, we used an R package ‘protr’ [64] to generate seven different features that have been extensively used in previous works. These features represent major compositional and physicochemical characteristics of a sequence and are described below:

#### 4.2.1. Amino Acid Composition (AAC)

The AAC of a protein sequence represents the fraction of each of the 20 standard amino acid residues. AAC has a fixed length of 20 features, and it can be mathematically represented as

(1) $$AAC\left(i\right)=\frac{AA_{i}}{K}$$

where *AA_i_* represents the number of amino acids of type *i*, and *K* denotes protein sequence length.

#### 4.2.2. Autocorrelation (AutoC)

Autocorrelation descriptors are defined based on the distribution of amino acid properties along the sequence. AutoC descriptors are grouped into three types: (i) Moran, (ii) Moreau-Broto, and (iii) Geary, and can be denoted by Equations (2)–(4), respectively.

(2) $$AC\left(d\right)=\overset{N-d}{\underset{i=1}{\sum }}P_{i}P_{i+d}d=1,2,\ldots ,nlag$$

where *d* is the lag of autocorrelation; *P_i_* and *P*_*i*+*d*_ are the amino acid properties at position *i* and *i*+*d*; nlag represents the maximum value of the lag.

(3) $$I\left(d\right)=\frac{\frac{1}{N-d}\Sigma_{i=1}^{N-d}\left(P_{i}-\bar{P}\right)\left(P_{i+d}-\bar{P}\right)}{\frac{1}{N}\sum_{i=1}^{N}{\left(P_{i}-\bar{P}\right)}^{2}}d=1,2,\ldots ,30$$

(4) $$C\left(d\right)=\frac{\frac{1}{2\left(N-d\right)}\Sigma_{i=1}^{N-d}{\left(P_{i}-P_{i+d}\right)}^{2}}{\frac{1}{N}\sum_{i=1}^{N}{\left(P_{i}-\bar{P}\right)}^{2}}d=1,2,\ldots ,30$$

where *d* is the autocorrelation lag, *P_i_* and *P_i+d_* are the amino acid properties at positions *i* and *i+d*, and $\bar{P}$ is the average value of property *P* denoted as: $\bar{P}=\sum_{i=1}^{N}P_{i}/N$.

#### 4.2.3. Composition (C), Transition (T), and Distribution (D) (CTD)

The CTD descriptors were described more than two decades ago to predict protein folding classes and represent the distribution of amino acid patterns for specific structural and physicochemical properties of protein sequences [65,66]. In CTD, the 20 standard amino acids are divided into three groups based on seven different types of physicochemical properties such as hydrophobicity, normalized van der Waals volume, polarizability, polarity, etc. (Table S2). In CTD, C is the fraction of polar, neutral, and hydrophobic residues of a given protein sequence:

(5) $$C\left(a\right)=\frac{Z_{a}}{K},a\in \left\{neutral,polar,hydrophobic\right\}$$

*Za* is the number of amino acids of type *a* in the given sequence.

*T* computes the percentage frequency of a specific property of an amino acid progressed by another property:

(6) $$T\left(ab\right)=\frac{Z_{ab}+Z_{ba}}{K-1},a,b\in \left\{\left(polar,neutral\right),\left(neutral,hydrophobic\right),\left(hydrophobic,polar\right)\right\}$$

where *Z_ab_* and *Z_ba_* represent the number of dipeptides encoded as *ab* and *ba* in the sequence.

Finally, D comprises five values for each of the three groups and determines the percentage of a target sequence length within which 25, 50, 75, and 100% of the amino acids of a specific property are located. In summary, CTD generates a feature vector of 147 dimensions.

#### 4.2.4. Conjoint Triad (CTriad)

The CTriad encodings were first used to predict protein-protein interactions [67]. In CTriad, a protein sequence is depicted as a vector space containing features of amino acids. Consequently, the vector space is trimmed by clustering the 20 naturally occurring amino acids based on their dipoles and side chains volumes, resulting in a 343-dimensional feature vector for any given protein sequence.

#### 4.2.5. Dipeptide Composition (DPC)

DPC is a fixed length of 400 (20 × 20) features and is defined as the frequency of two amino acid types in a given protein sequence:

(7) $$DPC\left(ab\right)=\frac{z_{ab}}{K-1}$$

#### 4.2.6. Quasi-Sequence Order (QSO)

QSO descriptors are derived by measuring the physicochemical distance between the amino acids of a given protein sequence and result in a fixed length of a 100-dimensional feature vector [68,69]. The first 20 quasi-sequence-order descriptors are defined as:

(8) $$X_{r}=\frac{f_{r}}{\sum_{r=1}^{20}f_{r}+w\sum_{d=1}^{maxlag}\tau d}\;r=1,2,\ldots ,20\;$$

where *f_r_* is the normalized occurrence for amino acid type, *I* and *w* is a weighting factor (*w* = 0.1). The other 30 quasi-sequence-order are defined as:

(9) $$X_{d}=\frac{w\tau_{d-20}}{\sum_{r=1}^{20}f_{r}+w\sum_{d=1}^{maxlag}\tau d}\;d=21,22,\ldots ,30\;$$

#### 4.2.7. Sequence Order Coupling Number (SOCN)

The *d*-th rank sequence-order-coupling number is defined as:

(10) $$\tau_{d}=\sum_{i=1}^{N-d}{\left(d_{i,i+d}\right)}^{2}d=1,2,\ldots ,maxlag$$

where *d*_*i*,*i*+*d*_ is the maximum lag, and the protein length must not be less than max lag.

### 4.3. Machine Learning Models

To get a quick approximation of the best ML classifier, we assessed five commonly used ML algorithms, namely, K-nearest neighbors (KNN), naive Bayes (NB), random forest (RF), support vector machines (SVM), and neural network (NNET), by using CARET [63]. Using the 10-fold cross-validation (CV) approach, we assessed the performance of a given set of eight feature encodings (AAC, AutoC, CTD, CTriad, DPC, QSO, SOCN, and Hybrid) using default parameters for each corresponding ML algorithm.

### 4.4. Feature Selection

To improve the feature representation capability and determine the subset of ideal features that can correctly classify C10 peptidase (streptopain) and non-C10 peptidase (non-streptopain) sequences, we used the R implementation of the Boruta package (v7.0.0) [70]. Boruta is a feature selection algorithm and feature ranking based on the RF algorithm. Boruta analyzes the feature importance values calculated for the real predictor variables against the shadow variables (i.e., variables created by the permutation of these variables across observations). For each run, an RF is trained using a double length set of predictor variables comprising of an equivalent number of actual and shadow variables. For each of the real predictor variables, a statistical test is performed comparing its significance in relation to the utmost importance value accomplished by a shadow variable. Each variable can be classified as important or unimportant based on the importance values. Finally, all unimportant and shadow variables are eliminated. The process is repeated until all variables have been classified as important or unimportant, or a specific number of runs (maxRuns) have been achieved [71]. The default value of the maxRuns parameter is 100, and we observed that it was too small for the algorithm to classify variables as important or unimportant. Therefore, we set the max runs parameter to 1000. Any remaining tentative features were checked by the TentativeRoughFix function, which compares the median Z-score of a tentative feature and the median of maximum Z-scores among shadow features (MZSF) across the previous RF runs and eventually makes a decision. Overall, Boruta performs a top-down approach for relevant features by comparing the set of original attributes [72] and has been used in many feature selection tasks [71,73,74].

### 4.5. Performance Evaluation Metrics

To estimate the performance of our ML models, we used four widely used metrics that estimate the quality of binary classification. These include sensitivity, specificity, accuracy, and Matthews’ correlation coefficient (MCC) and are expressed as:

$$Sensitivity=\frac{TP}{TP+TN}$$

$$Specificity=\frac{TN}{TN+FP}$$

$$Accuracy=\frac{TP+TN}{TP+FP+TN+FN}$$

$$MCC=\frac{\left(TP*TN\right)-\left(FP*FN\right)}{\sqrt{\left(TP+FP\right)\left(TP+FN\right)\left(TN+FP\right)\left(TN+FN\right)}}$$

where *TP*, *TN*, *FP*, and *FN* represent the true positive, true negative, false positive, and false negative, respectively. In all cases, the higher the value, the better the prediction performance.

## 5. Conclusions

Cysteine peptidases that belong to the C10 family are represented by streptopain or streptopain-like proteases. These enzymes are critical virulence factors that cause tissue damage and severe lethal effect in GAS-infected mice, involved in toxic shock syndrome and apoptosis. Initially identified in all GAS, this protease has been identified in several other bacterial species. Therefore, an attempt was made to construct a novel ML model (C10Pred) using SVM and optimal features from the primary amino acid sequences. The predictive performance of C10Pred on 10-fold cross-validation and three independent datasets (VS1, VS2, and VS3) exhibited encouraging performance. Our predictor is a handy tool to classify novel C10 family or streptopain-like proteins, and offers essential information for researchers interested in C10 family proteases.

## Figures and Tables

**Table 1:** A statistical summary of the training and independent datasets. * = Non-C10 family cysteine proteases; ** = All bacterial sequences except C10 family or streptopain proteins.

	Datasets			
Class	Training Set	Independent Validation Sets		
		VS1	VS2	VS3
Positive (C10 family cysteine proteases)	269	67	82	82
Negative	280	70	200 *	349 **

**Table 2:** Performance of SVM on various feature encodings in 10-fold cross-validation. Accuracy scores ≥ 90% are in bold.

Features	Dimension Size	Accuracy	Sensitivity	Specificity	MCC
AAC	20	0.880	0.874	0.886	0.759
AutoC	720	0.883	0.877	0.889	0.767
CTD	147	**0.900**	0.892	0.907	0.800
CTriad	343	0.878	0.874	0.882	0.756
DPC	400	**0.934**	0.944	0.925	0.869
QSO	100	0.889	0.885	0.893	0.778
SOCN	60	0.754	0.755	0.754	0.508
Hybrid	1790	**0.929**	0.944	0.914	0.858

**Table 3:** The best performance achieved by various feature encodings using optimal features on 10-fold cross-validation. Values in bold indicate improvement in performance by at least 2% after feature selection.

Features	Dimension Size	Accuracy	Sensitivity	Specificity	MCC
AutoC	102	**0.900**	0.881	0.918	0.800
CTD	89	0.907	0.903	0.911	0.814
CTriad	67	0.883	0.874	0.893	0.767
DPC	79	0.925	0.926	0.925	0.851
QSO	45	**0.913**	0.907	0.918	0.825
SOCN	58	0.756	0.755	0.757	0.512
Hybrid	139	**0.956**	0.944	0.968	0.913

**Table 4:** Performance comparison of various optimal feature encodings on VS1 dataset. Accuracy scores ≥ 90% are in bold.

Features	Accuracy	Sensitivity	Specificity	MCC
AutoC	0.839	0.731	0.943	0.692
CTD	0.839	0.791	0.886	0.681
CTriad	0.861	0.776	0.943	0.731
DPC	0.891	0.836	0.943	0.785
QSO	0.869	0.836	0.900	0.738
SOCN	0.737	0.701	0.771	0.474
Hybrid	**0.927**	0.896	0.957	0.855

## Data Availability

All data are available within this manuscript.

## Supplementary Materials

The following supporting information can be downloaded at: www.mdpi.com/article/10.3390/ijms23179518/s1.
