# Supplementary material for: C10Pred: A First Machine Learning Based Tool to Predict C10 Family Cysteine Peptidases Using Sequence-Derived Features
Source: Int J Mol Sci. 2022 Aug 23;23(17):9518. doi: 10.3390/ijms23179518 (PMC9455582; doi:10.3390/ijms23179518)
Supplement: Supplementary file 1 [file ijms-23-09518-s001.zip › Supplementary Figures S1-S3.pdf]

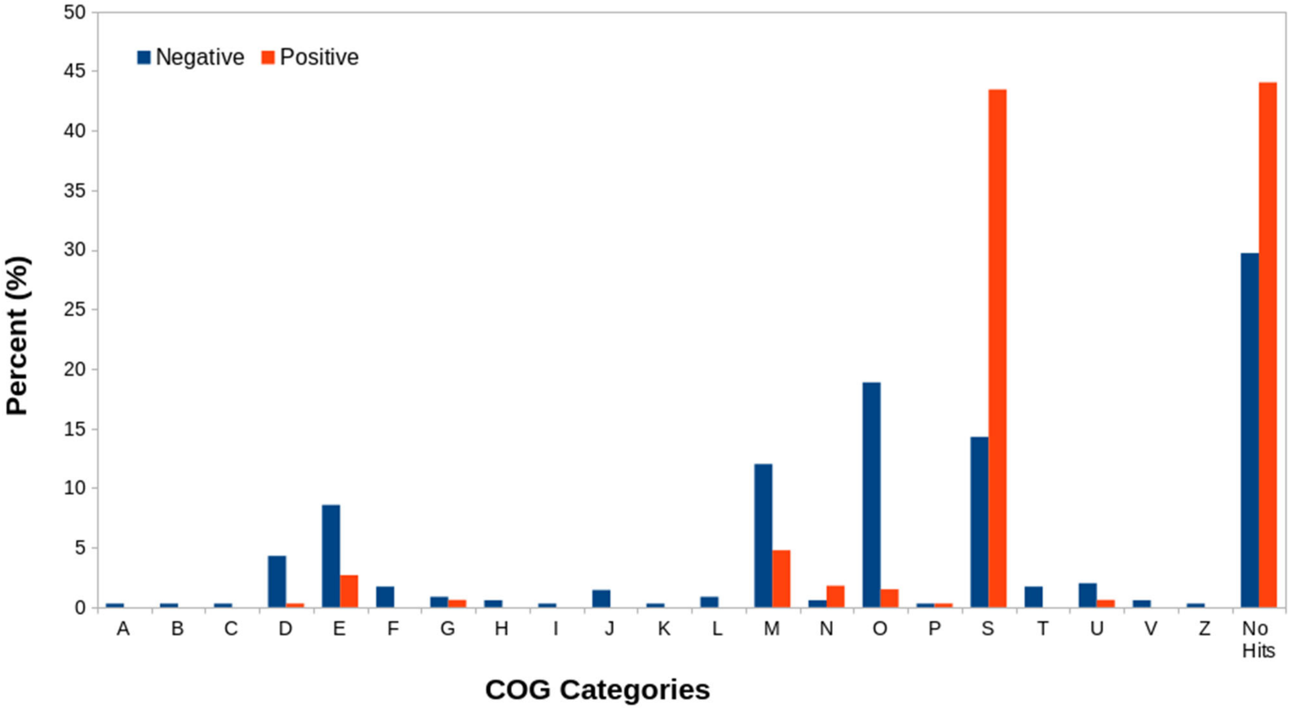

Figure S1: Distribution of various COG categories in positive and negative datasets

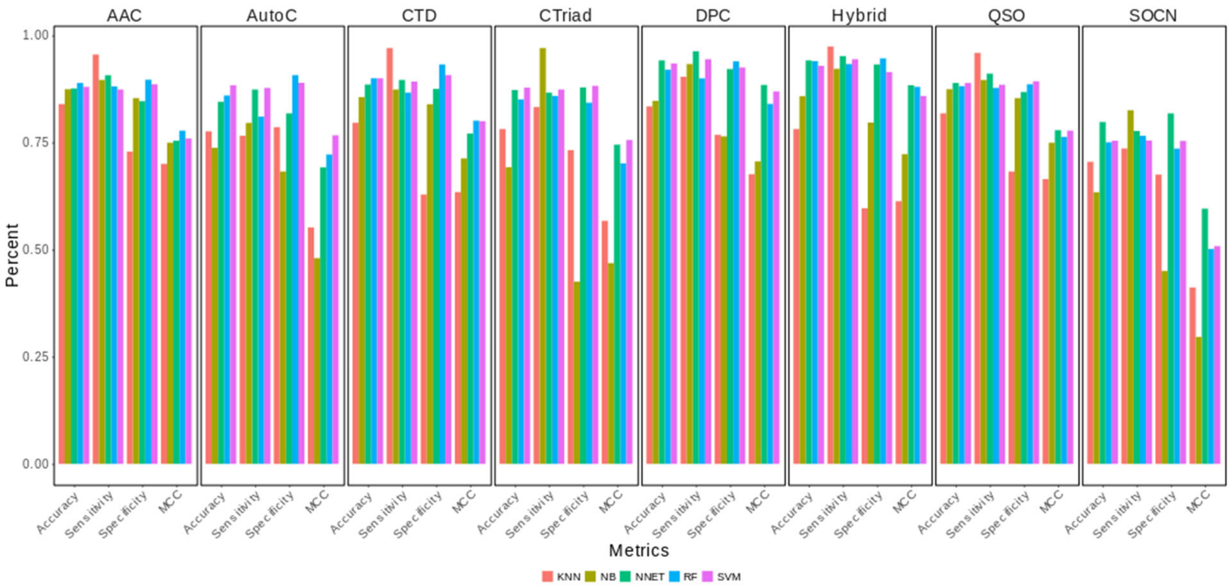

Figure S2: Performance comparison of four ML-based classifiers (KNN, NB, RF, SVM, and NNET) on seven different feature encodings and the hybrid.

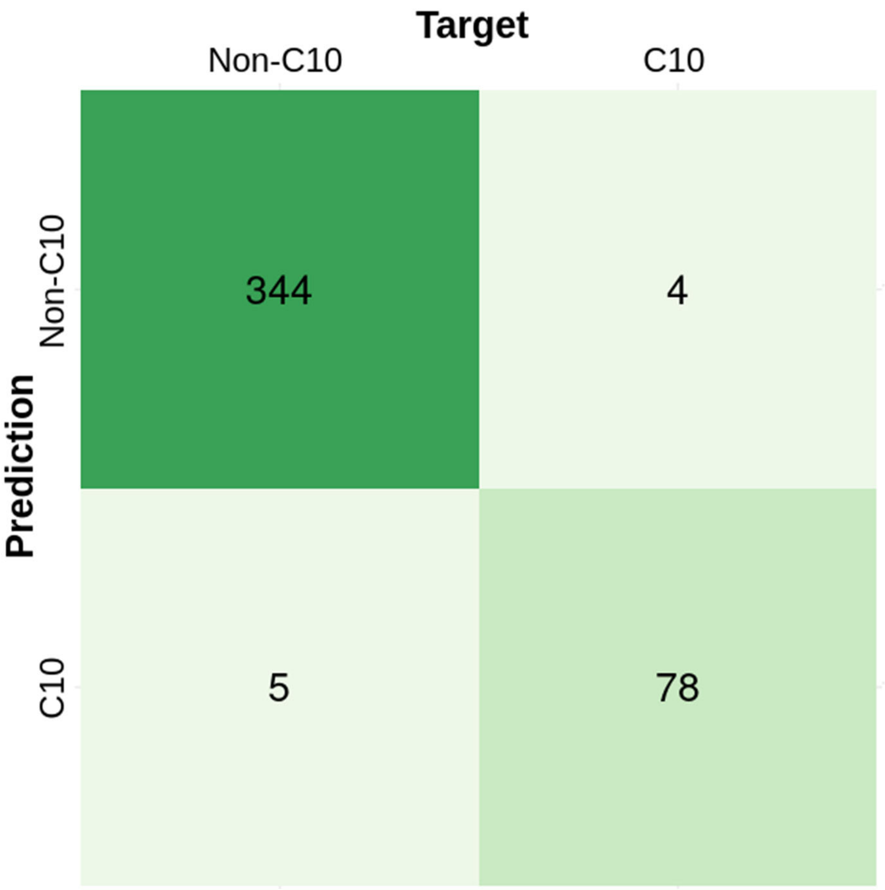

Figure S3: Confusion matrix of predicted results on additional independent dataset VS3. The matrix represents the distribution of output for each of the two classes (C10 or non-C10)
