# Supplementary material for: C10Pred: A First Machine Learning Based Tool to Predict C10 Family Cysteine Peptidases Using Sequence-Derived Features
Source: Int J Mol Sci. 2022 Aug 23;23(17):9518. doi: 10.3390/ijms23179518 (PMC9455582; doi:10.3390/ijms23179518)
Supplement: Supplementary file 1 [file ijms-23-09518-s001.zip › TableS2.pdf]

Table S2: Classification of 20 amino acids into three groups based on seven physicochemical properties

Property	Class I	Class II	Class III
Hydrophobicity	Polar<br>E, D, K, N, Q, R	Neutral<br>A, G, H, P, S, T, Y	Hydrophobicity<br>C, L, V, I, M, F, W
Normalized Van der Waals volume	0–2.78<br>A, C, D, G, P, S, T	2.95–4.0<br>E, I, L, N, V, Q	4.03–8.08<br>M, H, K, F, R, Y, W
Polarity	4.9–6.2<br>L, I, F, W, C, M, V, Y	8.0–9.2<br>A, G, P, S, T	10.4–13.0<br>H, Q, R, K, N, E, D
Polarizability	0–0.108<br>A, D, G, S, T	0.128–0.186<br>C, E, I, L, P, Q, V, N	0.219–0.409<br>K, M, H, F, R, Y, W
Charge	Positive<br>K, R	Neutral<br>A, N, C, Q, G, H, I, L, M, F, P, S, T, W, Y, V	Negative<br>D, E
Secondary Structure	Helix<br>A, E, H, K, L, M, Q, R	Strand<br>V, I, Y, C, W, F, T	Coil<br>D, G, N, P, S
Solvent Accessibility	Buried<br>A, C, F, G, I, L, V, W	Exposed<br>D, E, K, N, Q, R	Intermediate<br>M, S, P, T, H, Y
